# Supplementary material for: Body Mass Index Asian populations category and stroke and heart disease in the adult population: a longitudinal study of the Indonesia Family Life Survey (IFLS) 2007 and 2014
Source: BMC Public Health. 2023 Nov 10;23:2221. doi: 10.1186/s12889-023-17126-0 (PMC10636903; doi:10.1186/s12889-023-17126-0)
Supplement: Supplementary file 1 — Supplementary Material 1 [file 12889_2023_17126_MOESM1_ESM.docx]

**Supplementary Table S1. Incidence rate ratio of stroke and heart disease by** **BMI, stratified by living areas, age <55 years.**

|  | Body Mass Index categories for Asian population | | | | *P* value ^a^ |
| --- | --- | --- | --- | --- | --- |
|  | < 18.5 kg/m^2^ (underweight) | 18.5-22.9 kg/m^2^ (normal weight) | 23.0-27.4 kg/m^2^ (overweight) | ≥ 27.5 kg/m^2^ (obese) |  |
| Urban (n= 2,114) |  |  |  |  |  |
| No of participants | 123 | 695 | 786 | 510 |  |
| Stroke and heart disease, n (%) | 3 (2.4) | 25 (3.6) | 42 (5.3) | 36 (7.0) |  |
| Crude | 0.67 (0.20-2.24) | 1.00 (reference) | 1.48 (0.90-2.43) | 1.96 (1.17-3.26) | 0.028 |
| Model 1^b^ | 0.67 (0.20-2.23) | 1.00 (reference) | 1.51 (0.92-2.49) | 2.06 (1.22-3.47) | 0.038 |
| Model 2 ^c^ | 0.67 (0.20-2.23) | 1.00 (reference) | 1.52 (0.92-2.50) | 2.08 (1.23-3.40) | 0.031 |
| Model 3^d^ | 3.27 (0.59-17.95) | 1.00 (reference) | 2.85 (0.94-8.67) | 5.19 (1.69-15.85) | 0.016 |
| Model 4^e^ | 3.48 (0.63-19.18) | 1.00 (reference) | 2.93 (0.96-8.96) | 5.23 (1.70-16.06) | 0.049 |
| Rural (n= 2,163) |  |  |  |  |  |
| No of participants | 217 | 1,012 | 674 | 260 |  |
| Stroke and heart disease, n (%) | 2 (0.9) | 19 (1.8) | 25 (3.7) | 20 (7.6) |  |
| Crude | 0.49 (0.11-2.10) | 1.00 (reference) | 1.97 (1.08-3.58) | 4.09 (2.18-7.67) | <0.001 |
| Model 1^b^ | 0.47 (0.10-2.02) | 1.00 (reference) | 1.84 (1.01-3.38) | 3.67 (1.92-7.02) | <0.001 |
| Model 2 ^c^ | 0.45 (0.10-1.97) | 1.00 (reference) | 1.86 (1.01-3.40) | 3.71 (1.94-7.08) | <0.001 |
| Model 3^d^ | 0.46 (0.10-2.01) | 1.00 (reference) | 1.76 (0.96-3.24) | 3.27 (1.69-6.32) | <0.001 |
| Model 4^e^ | 0.46 (0.10-2.02) | 1.00 (reference) | 1.72 (0.93-3.16) | 3.23 (1.66-6.27) | 0.001 |
| ^a^ Poisson regression models.  ^b^ Model 1 was adjusted for sex.  ^c^ Model 2 was adjusted for model 1 plus region (Sumatra, Java-Bali, West Nusa Tenggara, Central, South and East Kalimantan, South, North and West Sulawesi).  ^d^ Model 3 was adjusted for model 2 plus employment status (employee or unemployment or missing) and education level (below lower-secondary or lower-secondary education and above or missing).  ^e^ Model 4 was adjusted for model 3 plus smoking status (current smoker or former smoker or non-smoker or missing), physical activity (yes or no or missing), visit to the health facility (yes or no or missing) and health insurance (yes or no or missing). | | | | | |

**Supplementary Table S2. Incidence rate ratio of stroke and heart disease by** **BMI**, **stratified by living areas, age ≥55 years.**

|  | Body Mass Index categories for Asian population | | | | *P* value ^a^ |
| --- | --- | --- | --- | --- | --- |
|  | < 18.5 kg/m^2^ (underweight) | 18.5-22.9 kg/m^2^ (normal weight) | 23.0-27.4 kg/m^2^ (overweight) | ≥ 27.5 kg/m^2^ (obese) |  |
| Urban (n= 1,117) |  |  |  |  |  |
| No of participants | 143 | 444 | 350 | 180 |  |
| Stroke and heart disease, n (%) | 12 (8.3) | 37 (8.3) | 23 (6.5) | 20 (11.1) |  |
| Crude | 1.00 (0.52-1.93) | 1.00 (reference) | 0.78 (0.46-1.32) | 1.33 (0.77-2.29) | 0.407 |
| Model 1^b^ | 1.04 (0.54-1.99) | 1.00 (reference) | 0.83 (0.49-1.40) | 1.43 (0.82-2.49) | 0.248 |
| Model 2 ^c^ | 1.05 (0.54-2.02) | 1.00 (reference) | 0.84 (0.49-1.42) | 1.43 (0.82-2.48) | 0.232 |
| Model 3^d^ | 1.01 (0.51-2.00) | 1.00 (reference) | 0.82 (0.48-1.40) | 1.39 (0.79-2.44) | 0.186 |
| Model 4^e^ | 0.94 (0.47-1.86) | 1.00 (reference) | 0.78 (0.46-1.34) | 1.30 (0.73-2.31) | 0.112 |
| Rural (n= 1,294) |  |  |  |  |  |
| No of participants | 291 | 669 | 247 | 87 |  |
| Stroke and heart disease, n (%) | 10 (3.4) | 30 (4.4) | 17 (6.8) | 13 (14.9) |  |
| Crude | 0.76 (0.37-1.56) | 1.00 (reference) | 1.53 (0.84-2.78) | 3.33 (1.73-6.38) | 0.002 |
| Model 1^b^ | 0.77 (0.37-1.58) | 1.00 (reference) | 1.59 (0.87-2.89) | 3.63 (1.87-7.04) | 0.002 |
| Model 2 ^c^ | 0.76 (0.37-1.56) | 1.00 (reference) | 1.60 (0.88-2.91) | 3.59 (1.85-6.96) | 0.004 |
| Model 3^d^ | 0.77 (0.37-1.58) | 1.00 (reference) | 1.55 (0.84-2.83) | 3.35 (1.70-6.59) | 0.010 |
| Model 4^e^ | 0.75 (0.36-1.54) | 1.00 (reference) | 1.51 (0.82-2.77) | 3.23 (1.69-6.43) | 0.030 |
| ^a^ Poisson regression models.  ^b^ Model 1 was adjusted for sex.  ^c^ Model 2 was adjusted for model 1 plus region (Sumatra, Java-Bali, West Nusa Tenggara, Central, South and East Kalimantan, South, North and West Sulawesi).  ^d^ Model 3 was adjusted for model 2 plus employment status (employee or unemployment or missing) and education level (below lower-secondary or lower-secondary education and above or missing).  ^e^ Model 4 was adjusted for model 3 plus smoking status (current smoker or former smoker or non-smoker or missing), physical activity (yes or no or missing), visit to the health facility (yes or no or missing) and health insurance (yes or no or missing). | | | | | |
